# The magic words: Using computers to uncover mental associations for use in magic trick design

**DOI:** 10.1371/journal.pone.0181877

**Published:** 2017-08-09

**Authors:** Howard Williams, Peter W. McOwan

**Affiliations:** School of Electronic Engineering and Computer Science, Queen Mary University London, London, United Kingdom; State University of New York Downstate Medical Center, UNITED STATES

## Abstract

The use of computational systems to aid in the design of magic tricks has been previously explored. Here further steps are taken in this direction, introducing the use of computer technology as a natural language data sourcing and processing tool for magic trick design purposes. Crowd sourcing of psychological concepts is investigated; further, the role of human associative memory and its exploitation in magical effects is explored. A new trick is developed and evaluated: a physical card trick partially designed by a computational system configured to search for and explore conceptual spaces readily understood by spectators.

## Introduction

With magic, as with most creative disciplines, there is little that is entirely new. Most creations are modifications, or syntheses, of existing artefacts (the tricks themselves) [1]. The process of designing a new magic trick often highlights aspects that could be automated or improved via a computational technique—work has been done to use computers as magic trick design aids, assisting with the creation of a card trick, and a magical jigsaw that exploits properties of the human visual perception system [2]. Here, a novel trick based on existing magical techniques is described, the creation of which has been aided by computational systems performing various tasks that would usually be performed by a human designer. The developed card trick, and the computational system used to help design it, rely on certain empirical observations, detailed and discussed below, about the way in which the human brain processes and reacts to language and imagery.

### Gilbreath principles

**Fig 1:**
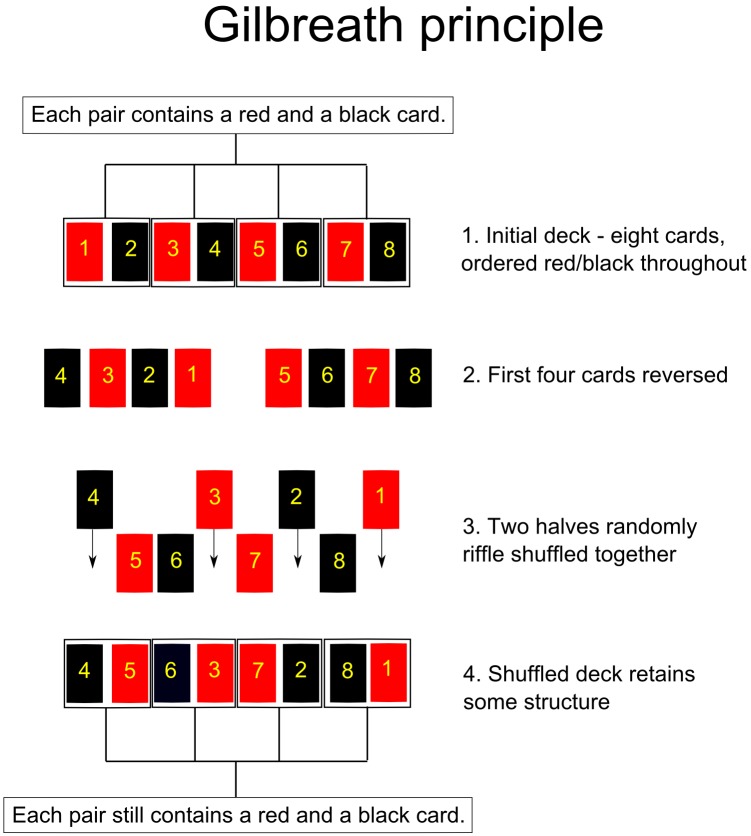
Gilbreath principle. An example of the Gilbreath principle. Eight cards are ordered red/black throughout. After reversing half the deck, and performing a riffle shuffle, each sequential pair still contains a red and a black card.

There are many known techniques available for use in the development of a new card trick; see Erdnase [3] and Hugard [4] for detailed discussions. Norman Gilbreath provided, in 1958, an ingenious set of observations about the mathematical properties of a deck of playing cards that magicians are able to exploit in numerous ways, commonly referred to as the Gilbreath [5] principles. These findings show that a deck of cards (or any sequence of objects) ordered in categorical groups, maintains, after one riffle shuffle, the property that all sequential groups in the deck are guaranteed to be composed of one of each card from each group, though not necessarily in the original order. To facilitate this prior to the shuffle, the order of one portion of the deck must be reversed. See Fig 1 for an example. Many card tricks detailed by, amongst others, Mactier [6], use these principles to great effect.

Card tricks sometimes rely on sleight of hand to manipulate cards that spectators have, supposedly secretly, selected, or force selection of a known card. A performer may skilfully keep track of cards in order to later, seemingly magically, reveal them. A classic type of effect is of the kind ‘select a card, any card’, which the performer then reveals. Essentially, this type of trick gives the participant the illusion of a free choice, which the performer is somehow able to divine. There are other ways to determine a spectator’s choices, that do not involve sleight of hand, which will now be discussed.

### Associative thinking

Mentalists (magicians concerned with the presentation of tricks that appear to rely on the workings of the human mind) sometimes rely on certain thought processes of their spectators to predict choices or behaviours. For example, a mentalist may ask a spectator to make a decision under time pressure, assuming that the decision making process will reduce to selecting prototypical mental representations. Banachek [7] describes a number of manipulations of this kind: “It’s your anniversary, and a messenger has just delivered a large box of flowers. What are they? Now!” Unsurprisingly, most people will name a rose in this situation. During the course of a trick, these predictions may err, though should this occur, the skilled conjurer will always have an alternative method, or even trick, lined up to save the situation. See Corinda [8], Earle [9], and Anneman [10] for discussions of this performance technique.

Mental objects—images, sounds, words, concepts, ideas—are often, in the cognitive sciences, termed representations: cognitive symbols that represent physical realities, or cognitive processes that make use of such symbols; see Von Eckardt [11] for detailed analysis. How one representation may give rise to another is a complex area of study for philosophers and psychologists. The so called Associationist school of thinkers believe that certain sensations, associated a sufficient number of times with certain ideas, may give rise to those same ideas by mere thought alone; see Hartley [12].

When magicians search for an as near as possible guaranteed association in the mind of a spectator, they look, knowingly or otherwise, for a particular property of the desired mental representations that will trigger the other: if one exists, the other exists [13]. Magicians would like strong associations such as those detailed in Pavlov’s famous experiment, see Shettleworth [14]: a dog was conditioned to associate the ringing of a bell with the appearance of food so strongly that an attendant response of salivation was produced on the ringing of the bell in the absence of food.

Implicit association is the idea that some concepts are subconsciously related in human minds—the strength of these automatic associations can be measured using the Implicit Association Test, presented by Greenwald [15]; a series of computer monitor based categorisation tasks, where speed of reaction is correlated to strength of association.

The human mind is a powerful associative machine. Representations can very easily be connected to one another, even when they are of different types. Magic tricks based on these kinds of mental association, such as the trick under discussion in this paper, can be seen as concrete instantiations of this type of theory of mental activity. The success, or otherwise, of the trick, may be seen as a kind of psychological test of the strength of the association of the mental objects deployed in the trick.

### Automatic thinking

Kahneman [16] has shown that the human mind appears to rely on two different psychological systems, which he terms System 1 and System 2. System 1, in Kahneman’s view, takes care of much of the seemingly automatic, yet sophisticated, mental processing that goes on in day to day life. A basic example of this in action, is the mental calculation required to evaluate the simple sum *x* in *x* = 2 + 3. This calculation, adding 2 and 3 together, happens so rapidly as to appear to our conscious minds as being an automatic process. Similarly automatically, the complex set of mental and physical processes required to pour some water into a glass and drink the contents is performed effortlessly, without error.

In contrast, consider calculating the value of the sum *x* in *x* = 373 + 259. This addition is easily calculable, with a little effort. The small amount of mental effort required to add the two numbers is an example of System 2 type thinking: active, conscious, applied thought for problems such as calculation, or planning. System 2 is the type of thinking that is able to, for example, solve puzzles by way of rational, contemplative thought. The same type of thinking can be applied by a spectator witnessing a magic trick, and may lead them to an understanding of the underlying method, spoiling the effect. It is this type of thinking that a magician will want to minimise during a performance. Equally, a performer will want to maximise the amount of System 1 type automatic thinking, as it is far more easily misled. Kahneman shows that given a choice between deploying the two systems to solve a given problem, most people will be comfortable accepting the immediately available solution presented by System 1.

### The trick

A mind reading prediction effect reliant on a set of custom playing cards is presented here. The trick has been designed with the assistance of a computational system configured with psychological constraints derived from the kind of observations of associative and automatic thinking discussed above.

During the performance of the trick the spectator is asked to make a seemingly free choice between certain presented options. After a card has been selected, the performer is able to reveal that this choice had been previously predicted by them.

To achieve this effect, the performer uses a physical set of playing cards that can be manipulated according to the Gilbreath principles. Further, Kahneman’s observations around System 1 thinking are built into the presentation of the trick, to engineer a situation for a participant whereby they will be asked to quickly make a choice between some associative options presented to them—in doing so, applying a kind of psychological force.

For ease of reference, the trick will be referred to as the **Association** trick. In a magic book, it could be described as:

From two shuffled decks of cards, the spectator freely chooses a word and a related image, which the performer seems to have been able to predict in advance.

#### 0.0.1 Template for the Association trick

The trick uses two decks of custom playing cards. One deck contains 16 distinct images, the other 16 distinct words, one per card. The words and images are derived from pre-defined, crowd sourced, conceptual categories. In each deck there are four separate categories, with four images, or four words, in each.

The underlying mechanism of the trick is that, in all, there are in fact only seven distinct conceptual categories. There is one further category that is deployed through both the deck of words and the deck of images. Note the fundamental point that there is one category that appears in both decks; all other categories are represented in either the deck of words, or the deck of images. The trick performance relies on the spectator selecting a word, and then coupling it with a related image, selected from a conceptually similar category in the image deck. The various categories that are used are critical to the efficacy of the trick. Each category used belongs to an overarching super-category (or, *theme*), that unifies the distinct categories in some way, for example they are all well known businesses. An automated process has been developed that allows a computer to take over many of the trial and error design decisions in selecting strong associations and categories previously incumbent on a human designer.

Using a numerical digit 1 to 7, to denote a card from a given conceptual category, the cards in each deck are initially ordered as:

- Word deck: 1, 2, 3, 4, 1, 2, 3, 4, 4, 3, 2, 1, 4, 3, 2, 1
- Image deck: 1, 5, 6, 7, 1, 5, 6, 7, 7, 6, 5, 1, 7, 6, 5, 1

There are two things to note: first, that the sequential ordering is reversed halfway through each deck, and second, the presence of category 1 in each deck. The second Gilbreath principle (which generalises the first principle) states that any sequentially ordered set of objects will retain elements of structure after one riffle shuffle.

To be clear, a riffle shuffle is one set of random interleaving operations performed on two parts of a deck; a deck is split into two sections, and randomly shuffled back together once. Usually, in Gilbreath based tricks, a sequentially ordered deck is split by dealing any number of cards face down from the top of the deck, which reverses their order. These cards are then riffle shuffled back together with the remaining cards from the deck. See Diaconis [5] for further explanations and explorations of these principles.

In the Association trick, half the full deck of the 16 image or word cards is pre-reversed, as shown above. Crucially, the structure that remains in this total stack of image or word cards after one riffle shuffle is guaranteed to hold one card from each category in each set of cards of appropriate length (here, four cards sets) dealt from the deck, though the ordering is now unknown. For the Association trick this means that, if each deck, cards and images, is riffle shuffled, dealing groups of four cards from the Word deck will yield groups containing cards from the categories [1,2,3,4], in some order. Similarly, the Image deck will yield groups containing cards from the categories [1,5,6,7], in some order.

The setup of the Association trick is therefore to order the two decks by category as described. The performance of the Association trick then runs:

1. The performer welcomes the spectator, and asks for their name, checking that they would like to participate in a mind reading experiment. Using a pad of paper, the performer apparently notes down their name, using some pretence (e.g. ‘I’ll just note your name, sometimes it helps me connect with people if I write their name out, I don’t know why…’). The pad of paper is put away.
2. The performer produces the two decks of cards, explaining that they contain Words and Images.
3. To show that the Word deck contains words, the performer deals eight cards *face up* on to the table, then quickly fans the remaining cards for the spectator to confirm that they are all word cards. The face up half of the deck is placed face down on the table, next to the other half, also face down.
4. The performer asks the spectator to shuffle the deck by pushing the two halves together, in a random fashion (or, if the spectator is comfortable handling cards, to riffle shuffle the deck back together).
5. An identical procedure is performed with the Image deck.
6. The performer, emphasising that the decks are now randomised, deals four piles of four cards from each deck, face down onto the table, making eight piles in total, taking care to keep the piles of words and images clearly separated. Each pile of four cards is dealt sequentially from the deck, before the dealer moves to the next pile.
7. The performer asks the spectator to select one pile of words, and one pile of images.
8. The performer now states that the spectator’s task is to quickly choose, from the eight cards in their hand, one word and one image that ‘go really well together; a good, strong match’, and to put the pair face up on the table. The intention is to put very mild psychological pressure on the spectator to make a quick, System 1, decision, rather than allowing their minds to have time to deploy System 2 type thinking, that may lead to idiosyncratic associations to be made between the cards.
9. The performer can appear interested in the selection at this point. The most likely choice that the spectator will have made is a word from category 1, with a matching image from category 1. All the other categories have been carefully chosen to be quite distinct from one another, though still related in some way to the theme, and so to all the words and images in each deck.
10. The performer now retrieves the pad of paper from the beginning of the trick, and reveals that, in addition to the spectator’s name, they also wrote a prediction about the cards they would choose. For example, if category 1 contains weather related images and words, a spectator may have chosen a picture of the sun, and the word ‘Rain’, and the performer could have written on the pad, about a spectator named Fred: ‘Fred is interested in the weather today’.

At the conclusion of the trick, the spectator should feel that the performer has impossibly predicted a totally free choice they have made about some random shuffled up words and images. The spectator recalls it was them that shuffled the cards, and made a free choice about which of the smaller dealt out piles of cards to use, and also the final pairing of cards.

What has actually happened is that the performer knows that, due to Gilbreath, at the end of the initial shuffling process the spectator will have a pile of images and words guaranteed to contain one word and one image from category 1 (and no more). The performer also knows in advance that the spectator should make a quick association between any of the four words and any of the four images from category 1, in preference to mixing any of the other categories, for example a word from category 3 with an image from category 6. Selecting suitably distinct categories is therefore critical. There is of course a chance that the spectator makes an unpredictable association, ruining the effect. We will see how likely this is in practise.

### Psychological factors

As seen from the description of the Association trick, its effectiveness relies on the careful selection of categories. Crucially, these categories must be chosen to minimise conceptual overlap. For example, while Fruits and Vegetables are distinct categories, it is not impossible to imagine a spectator choosing a picture of a red apple to match with the word ‘Beetroot’. The key factor is to reduce the potential matches between categories, leaving one easy choice: our category 1. However, this category must not glaringly stand out amongst the other categories, for fear of raising the spectator’s suspicions that the cards have been manipulated in some way; while the choice must be the most natural choice, it must also be mixed in with other choices that feel viable prior to serious consideration.

#### 0.0.2 Theme: Trademarks

Trademarks were chosen as a *theme* that the Association trick could be built around for this proof of principle experiment. A theme can be seen as consisting of lists of categories; for example, the trademark theme consists of brands (‘Nike’, ‘Google’, ‘Coca-Cola’, etc). In addition to automatically giving each image and word in each deck an overall themed similarity (loosely: companies), choosing trademarks as a theme capitalises on the work done by brand builders to cleanly separate the types of associative thoughts about each brand any given person may have. These thoughts fall into conceptual spaces crafted by the marketeers, from which distinct conceptual categories can be constructed.

From these categories—essentially pools of words and images—seven can be selected for use in the trick. Selecting seven categories that are conceptually far apart from one another minimises the chances that a spectator will make an association between a word and an image across categories, making it easier to stay within category 1, as required by the performer.

The overall grouping effect may be quite subtle, depending on the words and images used, but may be strong enough to give the decks of cards a credible feeling of cohesion.

#### Conceptual spacing

Trademarks are powerful cultural symbols that provide a pre-stratified set of conceptual spaces; they are very carefully constructed by advertisers and marketeers to carve out a niche area of mental space. There is commonality between the words and images that people think of when they see the trademarks, and these words and images minimally overlap with others that refer to different trademarks. Obviously, there is commonality between overarching groups, dependent on the market space that companies operate in. For example, the Ford trademark is likely to trigger similar general associations about vehicles as those triggered by the Mercedes trademark; however, there may be more specific associations that do not overlap; perhaps ‘luxury’ for the Mercedes, and ‘affordable’ for the Ford.

**Fig 2:**
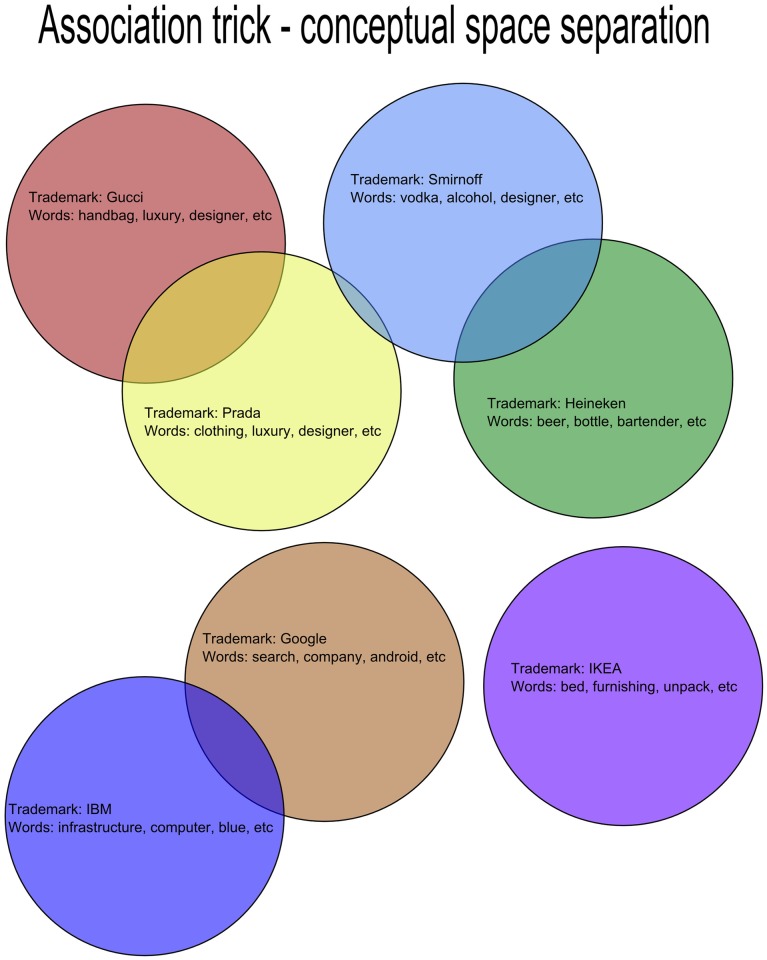
Conceptual spacing. The words that people use to describe certain trademarks allow the conceptual space around each to be defined. Some naturally group together, some are cleanly separated. The Association trick relies on the separated groups.

In addition to the words that are associated with each brand (via the trademark), there may also be common types of images (in addition to the trademark). This idea of conceptual space separation can be seen in Fig 2.

## Methods

### Psychological data bank

In order to determine a general view of trademarks in this way, an online experiment was run, in which participants (N = 87) were shown, in a random order, ten of the most famous one hundred trademarks, as determined by Millward Brown’s BrandZ [17] statement for 2013, in their annual review of the most well known brands from around the world. All one hundred brands/trademarks were covered, but each participant saw only ten. They were asked, for each trademark, to write words about how the trademark made them feel, or any associations at all that they had about the trademark, and also to make a line drawing of anything that they associated with the brand. The gathered responses form a kind of data bank of words and images that people call to mind when asked about trademarks.

These words and images can now be searched, categorised, and selected for deployment in decks of cards for use in the Association trick. The size of the data bank (870 distinct responses of words and images from the participants) makes it a difficult task for a human designer to sift through and group the various trademarks into conceptually distinct categories, and to pick out meaningful words and images for each category. This task can be performed computationally.

### Controlled problem domain

As noted, choosing the most conceptually distinct categories, and subsequently the words and images to populate each category, presents a challenge for the trick designer.

The data bank gained from the online trademark association experiment provides a series of queryable repositories; each trademark has a body of text associated with it, along with a series of images. Viewed in this way, it is possible to construct the problem of identifying categories of words and images from this heterogeneous data as an information retrieval problem: analysing data to find a set of words (or images) that best represent that data.

The main problem addressed here is the grouping of certain trademarks together into conceptual spaces based on the words used to describe them. The images gathered experimentally for the trademark theme provide a direct source for the human trick designer to use.

### Automated data gathering and processing

In addition to the automated identification of the best categories to use for the trick, the gathering of the data itself was also automated by a computer, reducing the need for direct psychological experiments to be performed. The power of search engines such as Google was harnessed to provide access to documents on the internet that belong to each class (e.g. trademarks/brands) of each theme. Instead of querying a human participant in an experiment to respond to trademarks using their own words, internet searches were performed—the web pages linked to by the top ten results for each trademark were then accessed and the words on the pages appended to the data bank repositories for the relevant trademark.

The problem faced by the Association trick designer is to group sets of similar classes from the data, for example Google and IBM, (to avoid having similar classes in different groups), and also to select words that belong to these classes and groups that are significant and meaningful.

The developed algorithm relies on the following computational concepts:

### Information content

Information content (IC) is a basic metric used in computational natural language processing to convey how specific a concept a word describes. Higher values indicate that a more specific concept is represented by a certain word (for example ‘pencil’ specifically describes a particular object that belongs to the more general conceptual group of writing implements); lower values indicate a more general concept (for example ‘idea’). The IC of a word can be computed in the context of a body of text; the more frequently occurring words are seen as having lower IC scores. The IC scores are used here as a text pre-processing tool—to reduce the number of words in the document store by pruning words with low IC scores (for example ‘the’, ‘and’, etc.). [18]

### Word similarity

A key process in computational language processing is to compare two words for semantic similarity. For example, the word ‘dog’ is semantically similar to the word ‘cat’, but not to the word ‘sky’. Providing a numerical measure of this kind of similarity is computationally difficult.

The WordNet system, originated by Miller [19], is a lexical database that describes hierarchical relationships between words, and is commonly used in natural language processing tasks. In WordNet, words are arranged into a tree structure that increases in specificity with depth; parent nodes subsume more specific instances—for example, the word ‘coin’ may be a parent to ‘penny’ and ‘pound’. WordNet provides a number of different similarity scoring mechanisms for two words, based on their parent nodes, and the depths of the respective words and parents. WordNet also provides sets of data describing synonyms for words.

More recently, work by Mikolov et al [20] [21] has produced a natural language processing tool called word2vec. The tool operates on datasets, learning vector representations of words using neural networks. The model is able to provide good word similarity scores.

### Okapi BM25 scoring

Information retrieval is a field of computer science dedicated to finding specified data in, often large, datasets. Okapi BM25 is a ranking function, first developed at London’s City University in the 1980s and 1990s for use in search engines [22] [23], that scores documents for relevance to a search query. ‘BM’ simply stands for ‘Best Match’, while ‘25’ reflects the function’s incremental development through BM11 and BM15 versions. Here, it is referred to as BM25.

It is feasible to perform internet searches to gather crowd sourced data about certain themes, that can then either replace or augment a document store derived experimentally. For the trademark theme, the document store was generated using a combination of these two methods.

#### 0.0.3 Association trick strong association selection algorithm

BM25 can be used by search engines to retrieve relevant documents from a document store, given a particular query. We use it slightly differently here. Viewing the generated data bank of words for each class in each theme as the document store, where each document refers to a particular class (e.g. ‘Nike’, for the trademark theme), it is possible to generate BM25 scores for each document in the document store, for each word in a given dictionary (using word2vec and WordNet for granular word similarity scoring).

These one word queries then have a set of ranked documents associated with them, which can be sorted with the highest scores at the top. Setting a threshold for the BM25 score, above which documents are seen as highly relevant to a particular query, allows the grouping of documents into classes defined by queries.

See Fig 3.

**Fig 3:**
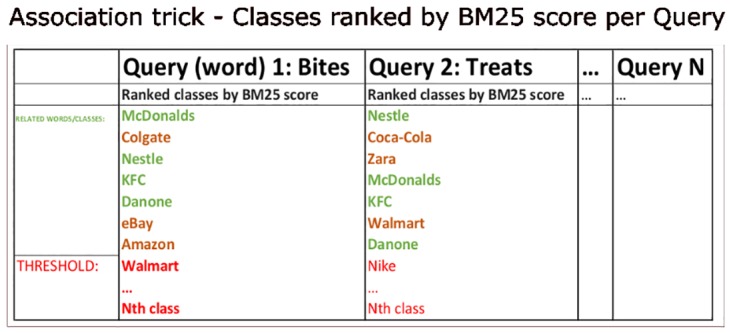
Document store processing. Simple examples of words (queries) with their associated classes (documents of words relating to a particular brand) ranked by BM25 score. Categories of classes can be picked out in groups, by filtering and merging the ranked lists. The green words are all closely related, and exist in both queries.

These scores also allow each document to be associated with multiple relevant queries. In this way, the document store can be categorised, and a set of words generated for each category (using BM25 scores for words in a dictionary used as queries to the documents for each category). This provides the trick designer with a pre-computed set of words for use in the Association trick.

A companion set of images may be generated by taking a set of words for this purpose and feeding them into an image search engine, or passing them to an artist. In the case of the trademark theme, empirically sourced images from experimental participants are available directly from the document store.

While the output so given will work, to generate the best trick possible, the human trick designer should still sift through the computer generated suggested items, picking out a further refined set. The computer acting as a form of computer assisted design tool.

**Fig 4:**
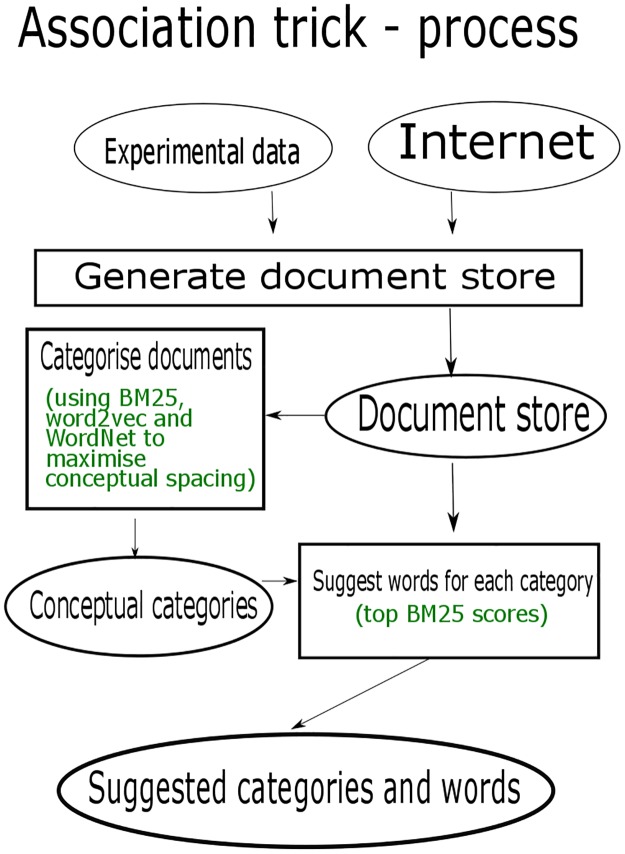
Generating the Association trick. The computational and experimental process for suggesting categories and words for use in the Association trick. The document store is sourced experimentally, and from the internet, before being processed and analysed for categories and words. If the theme is chosen well, the categories will naturally be conceptually far apart.

A visual representation of the process is shown in Fig 4.

## Results

### 0.0.4 Association trick algorithm outputs

The algorithm outlined is able to output suggested sets of categories, and words associated with these categories, which the trick designer may use to construct an Association trick. The benefit of using this automated system is that rapid prototypes of themed tricks may be automatically produced, which the trick designer is then able to fine tune, comparing different themes to each other to find a suitable set from which to produce a full trick.

This type of computational assistance is of the kind widely used in many creative areas such as music composition, photographic editing, and computer aided design, where the machine is seen as a useful creative assistant, rather than as a full blown creative entity. The human operator is still very much key to the most effective trick design process, though is now in possession of a powerful tool that can speed up the process, and potentially suggest ideas that may have been otherwise overlooked. Also the performers skills in presentation dramatically affect the overall magic.

### 0.0.5 Association trick algorithm computation time

The main factor that determines how long the algorithm takes to run is the number of combinations of categories to evaluate for semantic separation, from the generated category list. To evaluate each combination, on a computer with an Intel Core i5 processor, takes approximately:

$$\begin{array}{c} CategoryEvalTime=0.01\;seconds \end{array}$$ (1)

Allowing sets of seven categories (CategorySets) to be picked from the top 20 highest scoring categories (those with the most closely associated members: TopCategories), gives:

$$\begin{array}{c} CategoryCombinations=\frac{TopCategories!}{\left(TopCategories-CategorySets)!(CategorySets!\right)} \end{array}$$ (2)

$$\begin{array}{c} CategoryCombinations=\frac{20!}{\left(20-7)!(7!\right)}=77520, \end{array}$$ (3)

therefore, finding the set that are most conceptually distant takes approximately:

$$\begin{array}{c} RunTime=CategoryEvalTime\times CategoryCombinations=775.2\;seconds \end{array}$$ (4)

Given more time, a wider range of categories may be used (e.g. picking seven category combinations from a list of 30).

### 0.0.6 Suggested words

**Fig 5:**
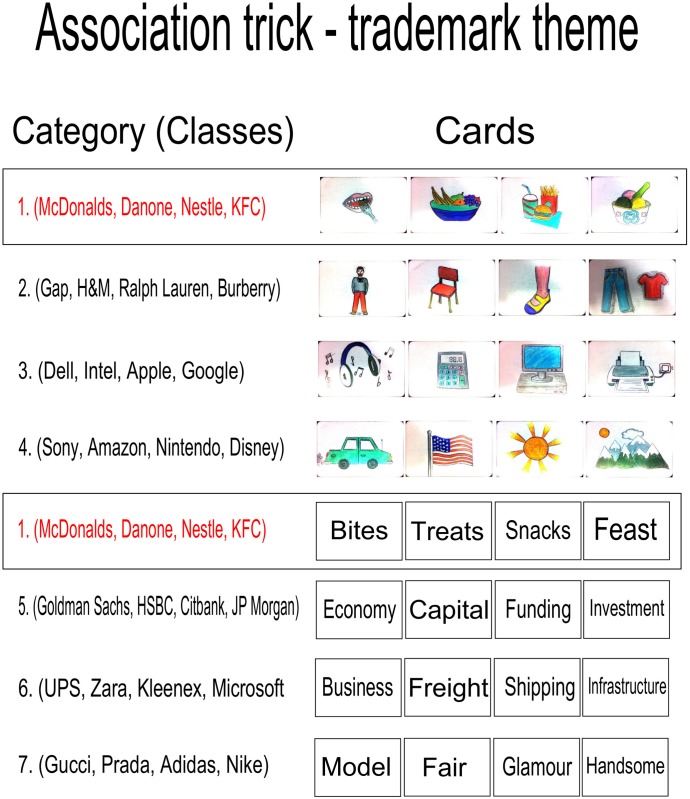
Trademarks. Cards produced for use in the Association trick, with a Trademark theme. Category 1 defines the cards that the performer hopes the spectator will match.

The algorithm was run for the trademark theme discussed, for 100 trademark classes, using a combination of the existing document store determined experimentally, and an internet sourced store. Seven categories were suggested from the top twenty identified categories. Words were manually selected (from the algorithmically suggested words) by the trick designer, and made up into a physical set of cards, that can be seen in Fig 5. The images were generated by an artist, using the experimentally determined document store of images for classes in the suggested categories, additionally informed by the suggested words from these classes.

The words suggested by the algorithm, selected by the trick designer, are more abstract than was anticipated, grouping classes of trademarks at quite high levels; some words are obviously directly related to certain members of the categories, e.g. ‘Shipping’ directly relates to ‘UPS’, a delivery company, while others only make sense on reflection: ‘Infrastructure’ relates to ‘Microsoft’ in the context of information technology infrastructure, and to ‘UPS’ in the context of a parcel delivery infrastructure. Some categories contain rather tenuously related classes and words; for example, ‘Kleenex’ and ‘Zara’ are both a ‘Business’, however, of course, all the classes in the trademark theme are businesses.

The use of more sophisticated semantic similarity word scoring techniques would improve results, and a more extensive data gathering exercise may allow the algorithm more meaningful options for suggestions. However, some categories are cleanly grouped: category 1 contains words that abstract various ideas around food that the trademarks it contains suggest, while the images provided from the empirically derived document store are strongly suggestive of the words, and vice versa; see Fig 5.

Something potentially quite nebulous about the group of trademarks in category 1 has been captured by the algorithm, that cleanly separates it from the other categories. While further pruning and improving of the decks of words and images could have been manually performed by a human designer, only suggestions made by the algorithm (and images in the document store) have been used to select from, in order to test the efficacy of the overall method.

### Evaluation of the trick

The Association trick was tested, with ratings given by participants, using the trademark theme cards shown in Fig 4, at a science fair: the Big Bang 2013, at the NEC in Birmingham, UK. The ratings were compared to the ratings from those gathered in a previous study for a set of classic magic tricks (known to be effective), reported in [2]. Participants in the Association trick experiment (N = 143) chose to sit down at a stall obviously marked as being about magic, and were thus likely self-selecting as being relatively interested in magic tricks. They were asked to take part in a science experiment that involved witnessing a trick, and then filling out a questionnaire that asked them to rate their enjoyment of the trick, to rate their enjoyment of magic in general, and also to describe their reactions to the Association trick, and to magic in general. This set-up enabled a ruse on which the denouement of the trick relies: writing down the name of the participant (‘I’ll just make a note of your name, for the data…’). In fact, the words that were written down were of the form: ‘[Mike] looks hungry!’, in anticipation of the participant selecting a word and image from category 1, which are all about food in some way.

This premise, that the participant will in fact choose an image and a word from category 1, is inherently risky. The free choice gives the trick some power; how, the spectator might wonder, can the performer predict a free choice? However, the associative machinery at work in a human mind does not always behave predictably. During testing at the science fair, the Association trick ‘failed’ 15 times out of 143. From these failures, it is interesting to note the word and image pairs that were selected by the participants: [Word: Model]-[Image: Clothes] (4), [Word: Model]-[Image: Car] (4), [Word: Handsome]-[Image: Clothes] (3), [Word: Glamour]-[Image: Clothes] (2), [Word: Funding]-[Image: Calculator] (2). In future iterations of the trick, these matches could be removed, either by modifying the algorithm to disallow certain terms, or by hand.

Successful performances of the Association trick received a mean rating score of 3.27 (out of 4), comparing favourably with the classic tricks. Participants in the Association trick experiment rated magic in general 3.50 (out of 4)—i.e., irrespective of their enjoyment of the Association trick, how much they enjoyed magic in general. It is to be expected that people’s rating of how much they enjoy a particular category of entertainment is likely to be higher than most particular instances in that category, as they will likely recall some of the finest examples when generalising. The key indicator, as previously defined by Williams and McOwan [2], is the difference between the score the trick receives, and the score the same group of participants give magic in general; for the Association trick this difference is is 0.23 (the closer to zero the better, negative scores are rare and exceptional), broadly in line with what is expected from a successful trick [2].

The qualitative view of the experience was recorded: the words chosen by the participants to reflect their experience of the trick. As previously, participants were asked to select as many words as they wished, from: Bored, Surprised, Obvious, Neutral, Impressed, Predictable, Amazed. The following word counts were received: Impressed (84), Surprised (40), Amazed (22), Predictable (7), Neutral (4), Obvious (1) and Bored (1).

Overall, it seems participants were mostly impressed by the performer’s ability to predict their choice. They were also surprised, and sometimes amazed; this general reaction of being impressed is interesting; it points to the trick being received well as a performance, and to being somewhat inexplicable; however, it also highlights that even though the trick scored highly from a numerical perspective, it is perhaps not received as a genuinely magical experience most of the time, rather the participants enjoy the experience, and are impressed that the performer has second guessed them, but possibly have some notion that the relatively elaborate setup of the trick points the way to the method.

This overall qualitative impression is reinforced when looking at the explanations given by the participants for how the trick works (when it succeeded). Often, a good explanation for how the trick worked was provided (often along with a high enjoyment rating, and some positive qualitative word selection). Of the 128 participants, 16 provided an essentially correct trick method. From these 16, the mean average rating is 3.0 (out of 4); still a good score, though lower than the overall average. This is to be expected; working out the method reduces participant’s enjoyment of magic tricks. The words used by the 16 were: Impressed (8), Surprised (6), Predictable (1), Neutral (1) and Obvious (1) (participants were free to select more than one word).

The most common suspicious moments reported were: writing at the beginning (20), shuffling of the cards (6), and the dealing of the cards (6). These provide good clues as to how to improve the presentation: a better mechanism may be required to make the prediction at the start of the trick, the spectators must always feel they have freely shuffled the cards (they have, in fact, but may in retrospect suspect they haven’t), and the dealing of the cards could be handled by the spectator. Most commonly, participants did not report any suspicious moments.

## Discussion

The Association trick has been described, and the computational design process followed has been detailed. This has highlighted issues around the complexity of configuring computers to work with sophisticated human constructs such as language, visual imagery, and mental associations. The computer has been shown to be a useful time saving tool, and to have value as a kind of suggestion device for a particular creative task. Natural language is difficult even for humans to be creative with, though here a method has been arrived at that allows the human designer overall creative control with the added benefit of being able to rely on a computational aggregator and data sourcing mechanism.

The Association trick is still very much a result of a human creative act, though a computer now stands in as a significant proxy for some of the process. Part of the optimisation of the trick, the conceptual separation and word/image selection, is assisted by a machine, resulting in a trick that was generally well received in the real world.

While the suggestions from the computer are often sub-optimal, and need to be filtered by a human, it is notable that other computational methods may be available, now and in the future, that perform better.

The process discussed highlights the inherent difficulties involved in designing tricks computationally; computers simply process information, and as yet have have no sense of what ‘works’ for real people; this capacity to deal with complex human factors in a trick, such as natural language, must currently be included in the system by the creative intervention of the trick designer. Relying on empirically sourced data to guide the algorithms has been shown to be essential; without the additional document store items sourced directly from people’s associative reactions to classes within a theme, the Association trick algorithm struggles to categorise classes from themes in strong, meaningful, useful ways, though is still able to make interesting suggestions about words associated to each class in the theme. Overall, the effect for spectators is magical, and has been brought about by the the blending of human and computational design processes.

## Supporting information

S1 Dataset Supporting results dataset. (XLSX)

S1 Datastore Supporting information datastore. (ZIP)
